# Importance of the Interaction between Heading Date Genes *Hd1* and *Ghd7* for Controlling Yield Traits in Rice

**DOI:** 10.3390/ijms20030516

**Published:** 2019-01-26

**Authors:** Zhen-Hua Zhang, Yu-Jun Zhu, Shi-Lin Wang, Ye-Yang Fan, Jie-Yun Zhuang

**Affiliations:** State Key Laboratory of Rice Biology and Chinese National Center for Rice Improvement, China National Rice Research Institute, Hangzhou 310006, China; zhangzhenhua@caas.cn (Z.-H.Z.); yjzhu2013@163.com (Y.-J.Z.); 15621566500@163.com (S.-L.W.); fanyeyang@caas.cn (Y.-Y.F.)

**Keywords:** flowering time, gene-by-gene interaction, *Hd1*, *Ghd7*, rice, yield trait

## Abstract

Appropriate flowering time is crucial for successful grain production, which relies on not only the action of individual heading date genes, but also the gene-by-gene interactions. In this study, influences of interaction between *Hd1* and *Ghd7* on flowering time and yield traits were analyzed using near isogenic lines derived from a cross between *indica* rice cultivars ZS97 and MY46. In the non-functional *ghd7*^ZS97^ background, the functional *Hd1*^ZS97^ allele promoted flowering under both the natural short-day (NSD) conditions and natural long-day (NLD) conditions. In the functional *Ghd7*^MY46^ background, *Hd1*^ZS97^ remained to promote flowering under NSD conditions, but repressed flowering under NLD conditions. For *Ghd7*, the functional *Ghd7*^MY46^ allele repressed flowering under both conditions, which was enhanced in the functional *Hd1*^ZS97^ background under NLD conditions. With delayed flowering, spikelet number and grain weight increased under both conditions, but spikelet fertility and panicle number fluctuated. Rice lines carrying non-functional *hd1*^MY46^ and functional *Ghd7*^MY46^ alleles had the highest grain yield under both conditions. These results indicate that longer growth duration for a larger use of available temperature and light does not always result in higher grain production. An optimum heading date gene combination needs to be carefully selected for maximizing grain yield in rice.

## 1. Introduction

Flowering time is a pivotal factor in the adaption of cereals to various ecogeographic environments and agricultural practices, which is controlled by an intricate genetic network. Florigens are at the core of the network, which are encoded by *Hd3a* and *RFT1* in rice [1,2]. The expression of *Hd3a* and *RFT1* are regulated by two important pathways mediating by *Hd1* and *Ehd1*, respectively [3]. *Hd1* has dual functions, which enhances florigen genes expressions under short-day (SD) conditions but inhibits florigen genes expressions under long-day (LD) conditions. The function conversion of *Hd1* is related to *PhyB*, *Se5*, *Ghd7* and *Ghd8* [4,5,6,7,8]. Function loss of any of these genes attenuates the conversion and maintains *Hd1* as an activator under any day-length conditions. *Ehd1* activates florigen genes expressions to promote flowering under both the SD and LD conditions [9]. *Ehd1* likely acts as a signal integrator, and its expression is regulated by many genes [3]. Recent studies revealed that *Hd1* represses expression of *Ehd1* through interaction with *Ghd7* or *DTH8* [6,7,8].

Flowering time is closely related to the grain yield for crop, owing to its key role in maintaining an appropriate balance between full use of resources and avoidance of environmental stresses. Many heading date (HD) genes were reported to affect yield traits, and their natural variations have been used in rice breeding, such as *Ghd7* [10], *DTH8*/*Ghd8* [11,12], *Hd1* [13,14], *OsPRR37*/*Ghd7.1*/*DTH7*/*Hd2* [15,16,17], *RFT1* [18] and *OsMADS51* [19,20]. Abiotic stresses during flowering, such as high temperature, low temperature, and drought, can pose a serious threat to spikelet fertility and consequently induce yield loss. The relationship between HD gene and abiotic stress has been given attention in recent years. The *Ehd1*-*Hd3a*/*RFT1* pathway responses stress signals mediated by *Ghd7* [21], *OsABF* [22] or *OsMADS51* [20]. They integrate low temperature, high temperature, and drought signals, respectively, into HD pathway, which induce or repress floral transition to avoid flowering in the stress environments. Moreover, *Ghd7* and other four HD genes, including *Ghd2* [23], *OsHAL3* [24], *OsWOX13* [25] and *OsJMJ703* [26], were found to be involved in drought or salt tolerance during vegetative phase.

When the pleiotropic effects of individual HD genes on yield traits have become recognized, the role of gene-by-gene interaction remains to be explored. In the present study, influences of *Hd1* and *Ghd7* on HD and yield traits were analyzed using near isogenic lines (NILs) and NIL-F_2_ populations derived from a cross between *indica* rice cultivars Zhenshan 97 (ZS97) and Milyang 46 (MY46). Our results showed that *Hd1* and *Ghd7* could independently promote and repress flowering, respectively, whereas the flowering-repressor function of *Hd1* under natural long-day (NLD) conditions required functional *Ghd7*. With delayed flowering, spikelet number and grain weight increased under both natural short-day (NSD) and NLD conditions, but the spikelet fertility and panicle number fluctuated. Rice lines with genotype of *hd1Ghd7* produced the highest grain yield under both conditions.

## 2. Results

### 2.1. Effects of Hd1 and Ghd7 on Heading Date

**Figure 1:**
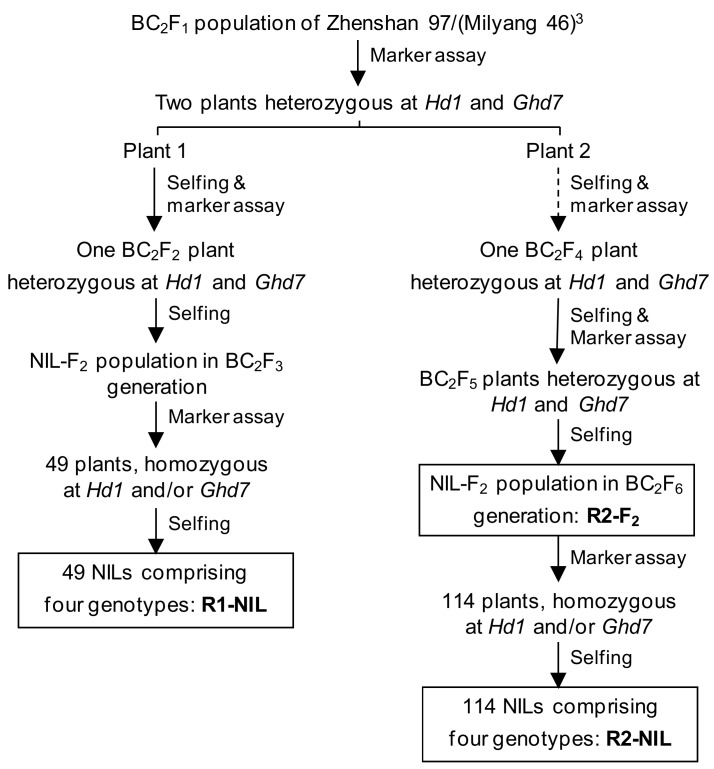
Development of the rice populations used in this study.

In this study, effects of *Hd1* and *Ghd7* on HD were investigated using three populations derived from the rice cross ZS97/MY46//MY46///MY46. ZS97 carries functional *Hd1* and non-functional *ghd7*, whereas MY46 carries non-function *hd1* and functional *Ghd7* [14,17]. The three populations included two NIL populations, namely R1-NIL and R2-NIL, and one NIL-F_2_ population namely R2-F_2_ (Figure 1). Each NIL population comprised all the four homozygous genotypic combinations of *Hd1* and *Ghd7*, i.e., *hd1*^MY46^*ghd7*^ZS97^, *Hd1*^ZS97^*ghd7*^ZS97^; *hd1*^MY46^*Ghd7*^MY46^ and *Hd1*^ZS97^*Ghd7*^MY46^. The NIL-F_2_ population consisted of all the nine genotypic combinations, i.e., *hd1*^MY46^*ghd7*^ZS97^, *Hd1*^heterozygous^*ghd7*^ZS97^, *Hd1*^ZS97^*ghd7*^ZS97^, *hd1*^MY46^*Ghd7*^heterozygous^, *Hd1*^heterozygous^*Ghd7*^heterozygous^, *Hd1*^ZS97^*Ghd7*^heterozygous^, *hd1*^MY46^*Ghd7*^MY46^, *Hd1*^heterozygous^*Ghd7*^MY46^, and *Hd1*^ZS97^*Ghd7*^MY46^. The R1-NIL population was tested under both the NSD and NLD conditions, and the R2-F_2_ and R2-NIL populations were tested in NLD conditions only. All the rice materials matured in seasons that are appropriate for rice growth.

The R1-NIL population consisted of 10, 7, 12, and 20 lines of *hd1*^MY46^*ghd7*^ZS97^, *Hd1*^ZS97^*ghd7*^ZS97^, *hd1*^MY46^*Ghd7*^MY46^, and *Hd1*^ZS97^*Ghd7*^MY46^, respectively. In the genetic background tested by whole-genome resequencing and marker analysis, this population was segregated at *Hd16* but homozygous at all the remaining 11 cloned quantitative trait loci (QTL) for HD, including *OsMADS51*, *DTH2*, *OsMADS50*/*DTH3*, *Hd6*, *Hd17*, *RFT1*, *Hd3a*, *OsPRR37*/*Ghd7.1*/*DTH7*/*Hd2*, *Hd18*, *DTH8*/*Ghd8* and *Ehd1*. The effects of *Hd1* and *Ghd7* on HD were tested under NSD conditions in Lingshui from Dec. 2016 to Apr. 2017 (16LS) and from Dec. 2017 to Apr. 2018 (17LS), and under NLD conditions in Hangzhou from May to Sep. in 2017 (17HZ).

**Figure 2:**
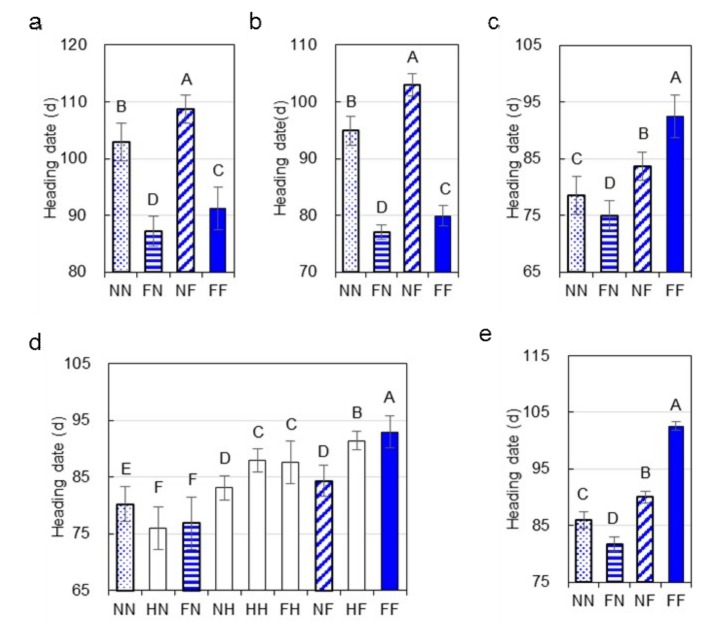
Heading date of rice lines classified based on the genotype of *Hd1* and *Ghd7*. (**a**) R1-NIL population under the NSD conditions in the 16LS trial. (**b**) R1-NIL population under the NSD conditions in the 17LS trial. (**c**) R1-NIL population under the NLD conditions the 17HZ trial. (**d**) R2-F_2_ population under the NLD conditions in the 17HZ trial. (**e**) R2-NIL population under the NLD conditions in the 18HZ trial. NN, *hd1*^MY46^*ghd7*^ZS97^; HN, *Hd1*^heterozygous^*ghd7*^ZS97^; FN, *Hd1*^ZS97^*ghd7*^ZS97^; NH, *hd1*^MY46^*Ghd7*^heterozygous^; HH, *Hd1*^heterozygous^*Ghd7*^heterozygous^; FH, *Hd1*^ZS97^*Ghd7*^heterozygous^; NF, *hd1*^MY46^*Ghd7*^MY46^; HF, *Hd1*^heterozygous^*Ghd7*^MY46^; FF, *Hd1*^ZS97^*Ghd7*^MY46^. Data are presented in mean ± sd. Bars with different letters are significantly different at *p* < 0.01 based on Duncan’s multiple range tests.

Highly significant effects (*p* < 0.0001) of *Hd1* and *Ghd7* on HD were detected in all the three trials (Table 1). In the two trials under NSD conditions (16LS and 17LS), the functional *Hd1*^ZS97^ and *Ghd7*^MY46^ alleles promoted and delayed flowering, respectively, no matter whether its counterpart was functional or non-functional (Figure 2a,b). In 16LS and 17LS, the proportion of phenotypic variance explained (*R*^2^) were estimated to be 80.74% and 75.69% for *Hd1*, and 5.79% and 6.50% for *Ghd7*, respectively. The interaction between *Hd1* and *Ghd7* was non-significant in the 17LS trial and significant in the 16LS trial with a small *R*^2^ of 1.30%. Overall, *Hd1* and *Ghd7* largely act additively in regulating HD under NSD conditions.

In the 17HZ trial under NLD conditions, the effects of *Hd1*, *Ghd7* and their interaction were all highly significant (*p* < 0.0001). The *R*^2^ were estimated to be 3.03% for *Hd1*, 56.54% for *Ghd7*, and 16.43% for the interaction between the two genes (Table 1). Compared with NILs having the *hd1*^MY46^*ghd7*^ZS97^ genotype, those having the *Hd1*^ZS97^*ghd7*^ZS97^ genotypes flowered earlier by 3.51 d; compared with NILs having the *hd1*^MY46^*Ghd7*^ZS97^ genotype, those having the *Hd1*^ZS97^*Ghd7*^MY46^ genotype flowered later by 8.75 d (Figure 2c; Table 2). These indicated that *Hd1* regulates flowering dependent on *Ghd7* under NLD conditions, and its flowering-repressor activity requires the functional allele of *Ghd7*. For *Ghd7*, it delays flowering regardless of genotype of *Hd1* but its effect is enhanced by *Hd1*. HD was longer by 5.24 d in lines of *hd1*^MY46^*Ghd7*^MY46^ than *hd1*^MY46^*ghd7*^ZS97^, whereas it was longer by 17.49 d in lines of *Hd1*^ZS97^*Ghd7*^MY46^ than of *Hd1*^ZS97^*ghd7*^ZS97^ (Table 2).

### 2.2. Expressions of Genes Involved in the Photoperiod Pathway

**Figure 3:**
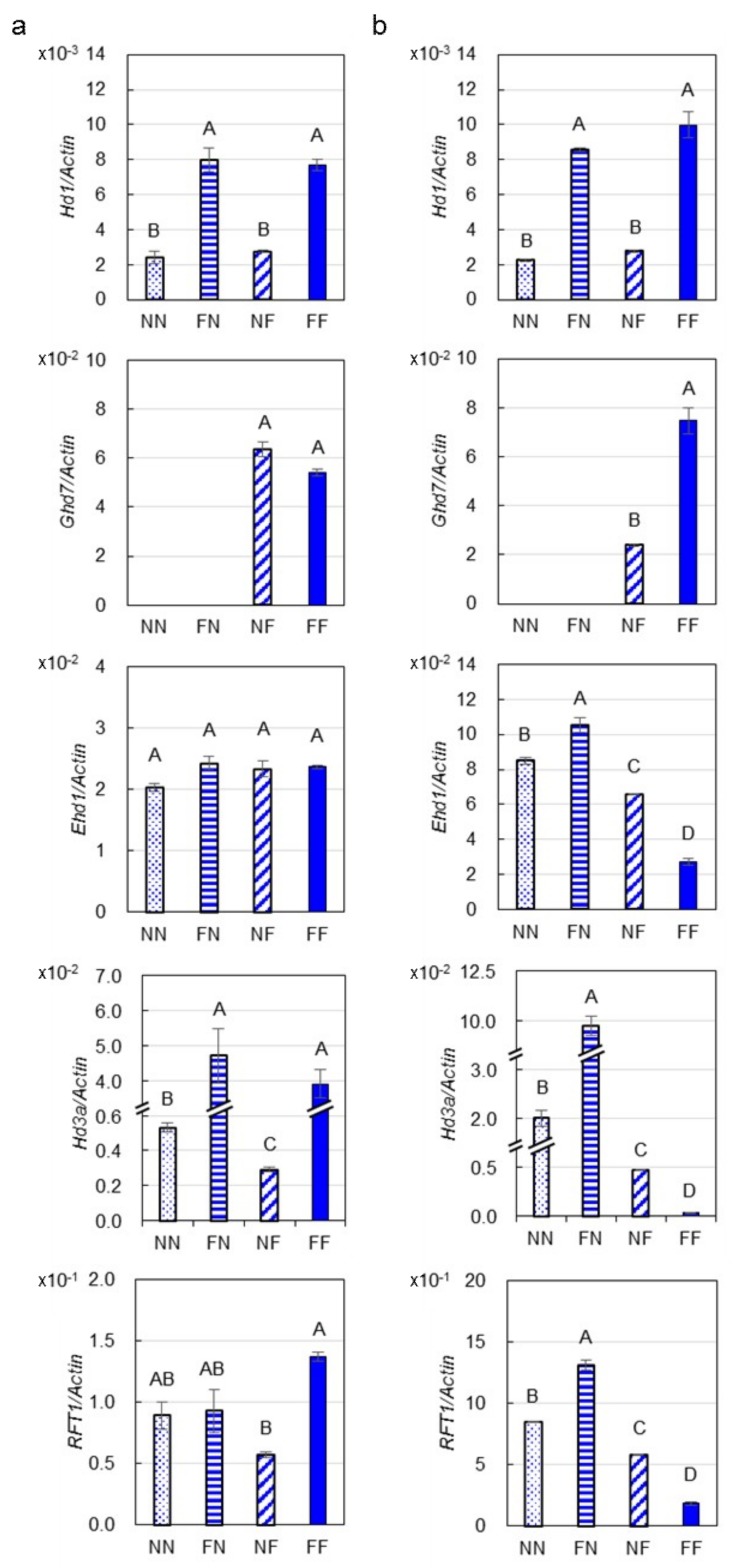
Transcript levels of five heading date genes in the R1-NIL population. (**a**) Under the NSD conditions in Lingshui. (**b**) Under the NLD conditions in Hangzhou. Data are presented in mean ± s. e. m (*n* = 3). Bars with different letters are significantly different at *p* < 0.01 based on Duncan’s multiple range tests.

The transcript levels of *Hd1*, *Ghd7*, *Ehd1*, *Hd3a* and *RFT1* at 2 h after sunrise were examined in seven-week-old rice lines in the R1-NIL population grown in the 17LS and 17HZ trials (Figure 3). In the 17LS trial under NSD conditions (Figure 3a), expression of *Hd1* and *Ghd7* was not affected by each other. The *Ehd1* expression was also not affected by either *Hd1* or *Ghd7*. For florigen genes, the expression of *Hd3a* was 7.87 times larger in lines of *Hd1*^ZS97^*ghd7*^ZS97^ than *hd1*^MY46^*ghd7*^ZS97^, and 12.46 times larger in lines of *Hd1*^ZS97^*Ghd7*^MY46^ than *hd1*^MY46^*Ghd7*^MY46^. These results indicate that *Hd1* promotes *Hd3a* expression regardless of *Ghd7* function, which was in accordance with that *Hd1* promotes flowering regardless of *Ghd7* function under NSD conditions. In addition, *Hd1* was also found to promote *RFT1* in the *Ghd7* background. At the same time, slightly repression of *Hd3a* by *Ghd7* was detected in the *hd1* background. These were consistent with the small effect of *Ghd7* under NSD conditions.

In the 17HZ trial conducted under NLD conditions (Figure 3b), expression of *Hd1* was not affected by *Ghd7*, but *Hd1* up-regulated *Ghd7* expression. The *Ghd7* expression was 2.12 times larger in lines of *Hd1*^ZS97^*Ghd7*^MY46^ than *hd1*^ZS97^*Ghd7*^MY46^. The expression of *Ehd1* in lines of *Hd1*^ZS97^*ghd7*^ZS97^ was 1.24 times as large as that in lines of *hd1*^MY46^*ghd7*^ZS97^, but the expression in lines of *Hd1*^ZS97^*Ghd7*^MY46^ was only 0.42 times as large as that in lines of *hd1*^MY46^*Ghd7*^MY46^. These suggest that *Hd1* significantly represses *Ehd1* expression in the *Ghd7* background. For florigen genes, the expressions of *Hd3a* and *RFT1* in lines of *Hd1*^ZS97^*ghd7*^ZS97^ were 4.86 and 1.55 times as large as that in lines of *hd1*^MY46^*ghd7*^ZS97^, indicating *Hd1* promotes expressions of florigen genes in the *ghd7* background. However, *Hd1* was converted to severely repress the florigen gene expressions in the *Ghd7* background. The expressions of *Hd3a* and *RFT1* in lines of *Hd1*^ZS97^*Ghd7*^MY46^ were only 0.07 and 0.32 times as large as those in lines of *hd1*^MY46^*Ghd7*^MY46^. In the meantime, significant repression of the *Ehd1*, *Hd3a* and *RFT1* expressions by *Ghd7* were detected in both the *Hd1* and *hd1* background, and the effect were larger in the *Hd1* background. The expressions of the three genes in lines of *hd1*^MY46^*Ghd7*^MY46^ were 0.77, 0.24 and 0.68 times as large as those in lines of *hd1*^MY46^*ghd7*^ZS97^; and the expressions in lines of *Hd1*^ZS97^*Ghd7*^MY46^ were 0.26. 0.004 and 0.14 times as large as those in lines of *Hd1*^ZS97^*ghd7*^ZS97^. These agreed with that flowering-repressor function of *Ghd7* could be enhanced by *Hd1*.

### 2.3. Influence of Hd1 and Ghd7 on Yield Traits and Its Relationship with HD

Grain yield per plant (GY), and five yield components traits including number of panicles per plant (NP), number of spikelets per panicle (NSP), number of grains per panicle (NGP), spikelet fertility (SF), 1000-grain weight (TGW), were measured in the R1-NIL population grown in the 17LS and 17HZ trials.

In the 17LS trial under NSD conditions, *Hd1* showed significant effects (*p* < 0.01) on all the six yield traits except NP; and *Ghd7* showed significant influences (*p* < 0.01) on all the six yield traits except SF and GY (Table 1). Interaction between the two genes were all non-significant at *p* < 0.01. Relationships between HD and the yield traits were further investigated (Table 2). The lines of *Hd1*^ZS97^*ghd7*^ZS97^ had the shortest HD, followed by *Hd1*^ZS97^*Ghd7*^MY46^, *hd1*^MY46^*ghd7*^ZS97^ and *hd1*^MY46^*Ghd7*^MY46^. Significant differences (*p* < 0.05) were detected for all the five yield determinants among the four genotypic groups. Three of the traits, NSP, NGP, and TGW, were positively correlated with HD, having correlation coefficients (*r*) of 0.823, 0.828, and 0.614, respectively (Table S1). Values of these three traits increased with delayed heading. On the other hand, NP and SF were not significantly correlated with HD. For GY, the values increased with delayed flowering among the three genotypic groups having the shortest to third shortest HD, and then remained stable when the HD became longer. Consequently, the two genotypic groups having the longest and second longest HD, *hd1*^MY46^*Ghd7*^MY46^ and *hd1*^MY46^*ghd7*^ZS97^, had little difference on GY.

In the 17HZ trial under NLD conditions, *Hd1* showed significant effects only on NP; and *Ghd7* showed significant influences on NP, NSP, and TGW (*p* < 0.0001). Significant interaction between the two genes was detected on TGW (*p* < 0.001). The interaction acted for increasing the values of the recombinant types, which was in accordance with the epistasis on HD. The HD and six yield traits were also compared among the four homozygous genotype groups (Table 2). The lines of *Hd1*^ZS97^*ghd7*^ZS97^ had the shortest HD, followed by *hd1*^MY46^*ghd7*^ZS97^, *hd1*^MY46^*Ghd7*^MY46^ and *Hd1*^ZS97^*Ghd7*^MY46^. Significant differences (*p* < 0.05) among the four genotypic groups were detected on four yield determinants, including NP, NGP, NSP, and TGW. Variations of TGW and NSP were positively correlated with HD, having *r* values of 0.708 and 0.355, respectively (Table S1). The two traits tended to increase with delayed heading. Similar tendency was observed for NGP though it was not significantly correlated with HD. Conversely, NP was negatively correlated with HD (*p* < 0.05), having *r* value of −0.670. SF also appeared to decrease with delayed heading though no significant difference was observed. Consequently, the largest value of GY in the four genotypic groups was observed for *hd1*^MY46^*Ghd7*^MY46^ which had the second longest HD.

### 2.4. Validation of the Influences of Hd1 and Ghd7 on HD and Yield Traits under NLD Conditions

The relationship between *Hd1* and *Ghd7* was further analyzed using the R2-F_2_ population, which was segregated at *Hd1* and *Ghd7* loci but homozygous at all the remaining 12 cloned flowering QTL mentioned above. The 775 plants of this population were grown in Hangzhou in 2017 under NLD conditions. Significant effects were identified for both genes. The additive effect, dominance effect and *R*^2^ were estimated to be 1.89 d, -0.89 d and 6.4% for *Hd1*, and 6.04 d, 1.91 d and 59.3% for *Ghd7*, respectively. The plants were classified into nine genotypic groups based on the *Hd1* and *Ghd7* alleles, and the HD values were compared (Figure 2d). *Hd1* promoted flowering in the *ghd7* background, but delayed heading when the genotype of *Ghd7* was functional or heterozygous. *Ghd7* delayed flowering regardless of the genotype of *Hd1* but its effect was enhanced by the functional *Hd1* allele.

Plants that were homozygous at *Hd1* and/or *Ghd7* were selected from the R2-F_2_ population and selfed. The resultant R2-NIL population, consisting of 29, 26, 29, and 30 lines of *hd1*^MY46^*ghd7*^ZS97^, *Hd1*^ZS97^*ghd7*^ZS97^, *hd1*^MY46^*Ghd7*^MY46^, and *Hd1*^ZS97^*Ghd7*^MY46^, respectively, was tested in Hangzhou in 2018 under NLD conditions. Both the *Hd1* and *Ghd7*, as well as their interaction, had highly significant effects (*p* < 0.0001) on HD (Table 1), which were similar to those observed previously under NLD condition. *Hd1*^ZS97^ promoted and repressed flowering in the *Ghd7*^MY46^ and *ghd7*^ZS97^ backgrounds, respectively, while *Ghd7*^MY46^ delayed flowering regardless of the *Hd1* function (Figure 2e).

GY and five yield components traits were also measured in the R2-NIL population. *Hd1* showed significant effects on SF (*p* < 0.0001) and GY (*p* < 0.01), and *Ghd7* exhibited highly significant effects on NSP, NGP, SF and TGW (*p* < 0.0001) (Table 1). Highly significant epistatic effects of the two genes were detected on all the traits except NGP (*p* < 0.0001). For NSP and TGW, the interactions acted for increasing the values of the recombinant types, which were consistent with the epistasis on HD. For NP, SF, and GY, the opposite direction was found. The relationships between HD and the yield traits were further analyzed (Table 2). Lines of *Hd1*^ZS97^*ghd7*^ZS97^ had the shortest HD, followed by *hd1*^MY46^*ghd7*^ZS97^, *hd1*^MY46^*Ghd7*^MY46^*, and Hd1*^ZS97^*Ghd7*^MY46^. Significant differences were detected for all the yield traits among the four genotypic groups. NSP, NGP and TGW were positively correlated with HD (*p* < 0.05), having *r* values of 0.806, 0.507 and 0.672, respectively (Table S1). Values of these traits increased with delayed heading. On the other hand, SF and NP were negatively correlated with HD (*p* < 0.05), having *r* values of −0.855 and −0.349, respectively. SF decreased with delayed heading; compared with lines having the shortest HD, SF in lines having the third longest, the second longest, and the longest HD decreased by 1.9%, 4.9% and 10.2%, respectively. Similar tendency was observed for NP. Consequently, lines in the *hd1*^MY46^*Ghd7*^MY46^ genotypic group having the second longest HD produced the highest GY.

## 3. Discussion

The bi-functional action of *Hd1* has been well recognized, promoting flowering under SD conditions and inhibiting flowering under LD conditions [27]. Recent studies revealed that flowering repressing function of *Hd1* is dependent on *Ghd7* [6,7]. In the present study, this relationship between *Hd1* and *Ghd7* was confirmed. Under NSD conditions, *Hd1* always up-regulated expressions of the two florigen genes (Figure 3) and promoted flowering regardless of *Ghd7* genotype (Figure 2). Under NLD conditions, *Hd1* still promoted flowering (Figure 2) by up-regulating florigen genes in the *ghd7* background (Figure 3). In the *Ghd7* background, however, *Hd1* was found to up-regulate *Ghd7*, and down-regulate *Ehd1* and florigen genes, consequently leading to late flowering. For *Ghd7*, its flowering-repressor action was observed under both NSD and NLD conditions regardless of *Hd1* function. Taken together, our results suggest that *Hd1* and *Ghd7* could promote and repress flowering independently, whereas flowering-repressor function of *Hd1* under LD conditions requires the functional *Ghd7*.

Among the four homozygous genotypic combinations of *Hd1* and *Ghd7*, the *Hd1ghd7* group exhibited the shortest HD under NLD conditions. Compared to *Hd1ghd7*, heading was delayed by 3.4–4.3 d and 7.5–8.7 d in the *hd1ghd7* and *hd1Ghd7* groups, respectively. Strikingly, HD in the *Hd1Ghd7* group was delayed by 16.1–20.9 d, owing to the genetic interaction between *Hd1* and *Ghd7* under NLD condition. This is likely the reason the *Hd1Ghd7* genotype was hardly carried by early season *indica* cultivars grown in middle-lower regions of the Yangtze River and South China regions [28] and *japonica* cultivars in northeast China [29], where early flowering is essential to ensure sufficient grown period for late season *indica* cultivars or secure a harvest before cold weather approaches.

It is generally accepted that long growth duration is associated with high-yielding production in rice [29,30], if varieties are harvested before cold weather approaches. A larger number of HD genes were found to have pleiotropic effects on yield traits, and their late-flowering alleles were frequently used to enhance grain yield mainly by increasing spikelet number and partially by increasing grain weight [10,11,12,13,14,15,16,17,18,19,20]. As expected, NSP and TGW gradually increased with delayed flowering under both the NSD and NLD conditions in this study. However, SF and NP tended to decrease under NLD conditions when the HD has become relatively long. As a consequence of trade-off among different yield components, rice lines having the *hd1Ghd7* genotype which had the second longest HD produced the highest grain yield, rather than the lines having the *Hd1Ghd7* genotype which had the longest HD. These results indicate that longer growth duration for a more use of available temperature and light does not always result in higher grain production.

Spikelet sterility is a key determinant of grain yield and frequently used as an indicator for stress tolerance. Two alternative explanations could be given to the decrease of spikelet sterility with delayed flowering. Firstly, alteration of time of flowering causes some loss of seasonal adaptability of rice. Secondly, *Ghd7* and *Hd1* participate in the stress tolerance of rice. *Ghd7* has been found to respond to multiple abiotic stress, such as high temperature, low temperature, and drought. Moreover, overexpression of *Ghd7* increases drought sensitivity, whereas knock-down of *Ghd7* enhances drought tolerance [21]. Our study showed that *Ghd7* expression was dramatically up-regulated in the *Hd1* background. This may be a reason that caused low SF in lines of *Hd1Ghd7*. Moreover, alteration of SF by *Hd1* was also observed in the *ghd7* background (Table 2), suggesting *Hd1* could be involved in stress response independently.

Panicle number is generally recognized as an unstable trait among yield traits. Few genes were reported to have pleiotropic effects on flowering time and panicle number [21,31,32]. *Ghd7* is found to regulate panicle number in a density-dependent manner. It decreases and increases panicle number at normal field condition and low-density conditions, respectively, though it always suppresses flowering time [21]. In the NIL populations used in our study, negative correlation between NP and HD was detected in both trials conducted under NLD conditions at normal planting density (Table 2, Table S1). The lines of *Hd1Ghd7* with the longest HD always produced the least NP (Table 2), indicating that combination of *Hd1* and *Ghd7* could cause decrease of panicle number under NLD conditions.

Although late-flowering alleles of flowering genes generally increase spikelet number, their influences on panicle number and spikelet sterility are not necessarily positive. Thus, an optimum HD genes combination needs to be carefully selected for maximizing grain yield in rice. In the present study, lines carried *hd1* and *Ghd7* alleles from MY46 produced the highest grain yield in both trials conducted in Hangzhou (Table 2) where is in the middle-lower region of the Yangtze River. Among the 14 middle-season *indica* rice cultivars tested by Wei et al [28], MY46 is one the 10 cultivars having the combination of non-functional *hd1* and functional *Ghd7*. These indicate that this combination could have undergone intensive artificial selection and play a significant role in the adaption of middle-season rice.

## 4. Materials and Methods 

### 4.1. Plant Material

Three rice populations segregating at both the *Hd1* and *Ghd7* loci were used in this study. The developing process was illustrated in Figure 1 and described below. One F_9_ plant of ZS97/MY46 was crossed with MY46 for two generations. Two BC_2_F_1_ plants which were heterozygous at both the *Hd1* and *Ghd7* loci were identified and selfed. In one of the two BC_2_F_2_ populations produced, a plant which was heterozygous for both the genes was identified and selfed. The resultant BC_2_F_3_ population was assayed with functional or closely linked DNA markers for the two genes. A total of 49 plants which were homozygous at *Hd1* and/or *Ghd7* loci were identified and selfed. One NIL population namely R1-NIL, comprising all the four homozygous genotypic combinations of *Hd1* and *Ghd7*, was constructed.

Another BC_2_F_2_ population was advanced to the BC_2_F_4_ generation. A BC_2_F_4_ plant which was heterozygous for both the genes was identified. In the resultant BC_2_F_5_ population, plants which were heterozygous for both the genes were selected and selfed. A NIL-F_2_ population in the BC_2_F_6_ generation, namely R2-F_2_ population, was constructed. A total of 114 plants which were homozygous at *Hd1* and/or *Ghd7* loci were selected and selfed. One NIL population namely R2-NIL, which consisted of all the four homozygous genotypic groups, was constructed.

### 4.2. Field Experiments and Phenotyping

The rice populations were tested in the experimental stations of the China National Rice Research Institute located at either Hangzhou or Lingshui. During the period of floral transition in the rice materials tested, day length in Hangzhou and Lingshui were corresponding to NLD and NSD conditions, respectively [14]. In all the trials, the planting density was 16.7 cm × 26.7 cm. Field management followed the normal agricultural practice. For NIL sets, the experiments followed a randomized complete block design with two replications. In each replication, one line was grown in a single row of ten plants. HD was recorded for each plant. At maturity, five middle plants in each row were harvested in bulk and measured for six yield traits, including NP, NSP, NGP, SF (%), TGW (g) and GY (g). Of which TGW was evaluated using fully filled grain followed the procedure reported by Zhang et al. [33].

### 4.3. DNA Marker Genotyping and Quantitative Real-time PCR Analysis

For population development and QTL mapping, total DNA was extracted using 2 cm-long leaf sample following the method of Zheng et al. [34]. PCR amplification was performed according to Chen et al. [35]. The products were visualized on 6% non-denaturing polyacrylamide gels using silver staining or on 2% agarose gels using Gelred staining. Three DNA markers were used, including functional marker Si9337 for *Hd1*, functional marker Se9153 and closely linked marker RM5436 for *Ghd7* [10,17].

For expression analysis, penultimate leaves of rice lines in the R1-NIL population were harvested at 7:00 am in 17HZ and 9:00 am in 17LS, 2 h after sunrise. Total RNA was extracted using RNeasy Plus Mini Kit (QIAGEN, Hilden, German). First-strand cDNA was synthesized using ReverTra AceR Kit (Toyobo, Osaka, Japan). Quantitative real-time PCR was performed on Applied Biosystems 7500 using SYBR qPCR Mix Kit (Toyobo, Osaka, Japan) according to the manufacturer’s instructions. *Actin1* was used as the endogenous control. The data were analyzed according to the 2^-Δ*Ct*^ method. Three biological replicates and three technical replicates were used. The primers were selected from previous studies [10,20,36].

### 4.4. Data Analysis

For the NIL-F_2_ population, QTL analysis was performed with single marker analysis in Windows QTL Cartgrapher 2.5 [37]. For the NIL populations, two-way ANOVA was conducted to test the main and epistatic effects. Duncan’s multiple range test was used to examine the phenotypic differences among genotypic groups. The analysis was performed using the SAS procedure GLM [38].

## Figures and Tables

**Table 1:** The effects of *Hd1* and *Ghd7* on heading date and six yield traits.

Population	Trial	Trait	*Hd1*			*Ghd7*			*Hd1* × *Ghd7*		
			*P*	*A*	*R*^2^%	*P*	*A*	*R*^2^%	*P*	I-effect	*R*^2^%
R1-NIL	16LS	HD	<0.0001	10.09	80.74	<0.0001	0.51	5.79	<0.0001	−1.30	1.30
	17LS	HD	<0.0001	7.95	75.69	<0.0001	0.77	6.50	0.2586		
		NP	0.5940			0.0014	−0.44	7.51	0.0103		
		NSP	<0.0001	7.57	35.99	<0.0001	4.35	20.36	0.0438		
		NGP	<0.0001	7.60	41.05	<0.0001	3.62	17.38	0.1490		
		SF	0.0015	0.94	10.18	0.7543			0.1061		
		TGW	<0.0001	0.99	51.36	<0.0001	0.32	11.31	0.0479		
		GY	<0.0001	3.26	45.02	0.0122			0.0575		
	17HZ	HD	<0.0001	−3.30	3.03	<0.0001	6.08	56.54	<0.0001	3.06	16.43
		NP	<0.0001	0.72	4.93	<0.0001	−1.08	20.84	0.3167		
		NSP	0.0442			<0.0001	5.45	13.87	0.2970		
		NGP	0.1371			0.0677			0.3892		
		SF	0.7773			0.0198			0.8471		
		TGW	0.9515			<0.0001	1.03	38.44	0.0002	0.46	3.49
		GY	0.4677			0.8271			0.5233		
R2-NIL	18HZ	HD	<0.0001	−2.34	6.60	<0.0001	6.15	62.57	<0.0001	4.20	28.27
		NP	0.8453			0.0304			<0.0001	−0.45	9.97
		NSP	0.0253			<0.0001	9.50	43.27	<0.0001	4.07	7.81
		NGP	0.8697			<0.0001	4.05	16.34	0.0610		
		SF	<0.0001	0.99	3.10	<0.0001	−3.25	46.28	<0.0001	−1.80	14.12
		TGW	0.5604			<0.0001	0.33	24.38	<0.0001	0.29	18.28
		GY	0.0097	0.66	2.68	0.6200			<0.0001	−1.14	7.33

16LS, the trial conducted under natural short-day (NSD) conditions in Lingshui from Dec. 2016 to Apr. 2017; 17LS, the trial conducted under NSD conditions in Lingshui from Dec. 2017 to Apr. 2018; 17HZ, the trial conducted under the natural long-day (NLD) conditions in Hangzhou from May to Sep. in 2017; 18HZ, the trial conducted under the NLD conditions in Hangzhou from Apr. to Aug. in 2018. HD, heading date; NP, number of panicles per plant; NSP, number of spikelets per panicle; NGP, number of grains per panicle; SF, spikelet fertility (%); TGW, 1000-grain weight (g); GY, grain weight per plant (g). *A*, additive effect of replacing a Zhenshan 97 allele with a Milyang 46 allele. *R*^2^%, proportion of phenotypic variance explained by the QTL effect. I-effect, positive value: parental type < recombinant type; negative value: parental type > recombinant type.

**Table 2:** Heading date and six yield traits of the four homozygous genotypes of *Hd1* and *Ghd7*.

Population	Trial	Group	HD	NP	NSP	NGP	SF	TGW	GY
R1-NIL	17LS	FN	87.2 ± 2.6 ^Dd^	11.8 ± 1.1 ^ABb^	79.3 ± 4.4 ^Cc^	69.3 ± 4.7 ^Cd^	87.4 ± 3.1 ^Bb^	25.4 ± 1.1 ^Cd^	20.7 ± 2.8 ^Cc^
		FF	91.2 ± 3.8 ^Cc^	11.6 ± 1.1 ^ABb^	87.9 ± 6.9 ^Bb^	77.6 ± 5.9 ^Bc^	88.3 ± 2.6 ^ABb^	27.0 ± 1.0 ^Bc^	24.0 ± 2.9 ^Bb^
		NN	103.0 ± 3.3 ^Bb^	12.6 ± 0.9 ^Aa^	92.0 ± 5.8 ^Bb^	83.3 ± 5.5 ^Bb^	90.6 ± 2.4 ^Aa^	28.2 ± 0.9 ^Ab^	29.4 ± 2.6 ^Aa^
		NF	108.6 ± 2.5 ^Aa^	11.0 ± 0.8 ^Bb^	108.2 ± 7.2 ^Aa^	96.7 ± 7.0 ^Aa^	89.3 ± 2.4 ^ABab^	28.9 ± 1.0 ^Aa^	29.9 ± 3.0 ^Aa^
	17HZ	FN	75.0 ± 1.6 ^Dd^	15.5 ± 1.2 ^ABa^	107.6 ± 8.6 ^ABb^	89.0 ± 5.7 ^Aab^	82.8 ± 3.9 ^Aa^	22.8 ± 0.8 ^Cb^	30.4 ± 1.5 ^Aa^
		FF	78.5 ± 2.1 ^Cc^	16.1 ± 1.2 ^Aa^	100.1 ± 6.8 ^Bc^	83.5 ± 4.0 ^Ab^	83.6 ± 3.9 ^Aa^	23.8 ± 0.8 ^BCb^	30.5 ± 1.8 ^Aa^
		NN	83.7 ± 1.5 ^Bb^	14.5 ± 1.2 ^BCb^	112.6 ± 9.1 ^Aab^	89.8 ± 9.1 ^Aab^	79.7 ± 5.7 ^Aa^	24.9 ± 1.1 ^ABa^	30.9 ± 3.1 ^Aa^
		NF	92.5 ± 1.6 ^Aa^	13.1 ± 1.4 ^Cc^	115.0 ± 10.7 ^Aa^	91.3 ± 11.8 ^Aa^	79.6 ± 8.5 ^Aa^	25.8 ± 1.2 ^Aa^	29.9 ± 4.5 ^Aa^
R2-NIL	18HZ	FN	81.6 ± 1.2 ^Dd^	12.6 ± 1.0 ^Aa^	115.4 ± 5.1 ^Dd^	105.3 ± 4.9 ^Bb^	91.3 ± 1.6 ^Aa^	25.0 ± 0.3 ^Cc^	31.8 ± 2.9 ^ABb^
		FF	86.0 ± 1.4 ^Cc^	11.7 ± 0.9 ^BCbc^	120.4 ± 6.7 ^Cc^	107.6 ± 5.9 ^Bb^	89.3 ± 2.1 ^Bb^	25.6 ± 0.4 ^Bb^	30.8 ± 2.9 ^Bbc^
		NN	90.0 ± 1.0 ^Bb^	12.2 ± 1.2 ^ABab^	131.5 ± 8.8 ^Bb^	113.5 ± 7.9 ^Aa^	86.4 ± 2.1 ^Cc^	25.7 ± 0.6 ^Bb^	33.3 ± 2.7 ^Aa^
		NF	102.5 ± 0.7 ^Aa^	11.3 ± 1.2 ^Cc^	142.7 ± 7.5 ^Aa^	115.7 ± 6.7 ^Aa^	81.1 ± 2.7 ^Dd^	26.3 ± 0.5 ^Aa^	29.8 ± 3.2 ^Bc^

FN, *Hd1*^ZS97^*ghd7*^ZS97^; FF, *Hd1*^ZS97^*Ghd7*^MY46^; NN, *hd1*^MY46^*ghd7*^ZS97^; NF, *hd1*^MY46^*Ghd7*^MY46^. Values are mean ± sd. Uppercase and lowercase letters following the values represent significant differences at *p* < 0.01 and *p* < 0.05, respectively, based on Duncan’s multiple range tests.

## Supplementary Materials

Supplementary materials can be found at http://www.mdpi.com/1422-0067/20/3/516/s1.

## Abbreviations

NSDs	Natural short-day conditions
NLDs	Natural long-day conditions
SD	Short-day conditions
LD	Long-day conditions
NIL	Near isogenic lines
ZS97	Zhenshan 97
MY46	Milyang 46
HD	Heading date
QTL	Quantitative trait locus
*R* ^2^	The proportion of phenotypic variance explained
GY	Grain yield per plant
NP	Number of panicles per plant
NSP	Number of spikelets per panicle
NGP	Number of grains per panicle
SF	Spikelet fertility
TGW	1000-grain weight
*r*	Correlation coefficient
