# Supplementary material for: Importance of the Interaction between Heading Date Genes Hd1 and Ghd7 for Controlling Yield Traits in Rice
Source: Int J Mol Sci. 2019 Jan 26;20(3):516. doi: 10.3390/ijms20030516 (PMC6387254; doi:10.3390/ijms20030516)
Supplement: Supplementary file 1 [file ijms-20-00516-s001.pdf]

Table S1. Correlation coefficients between heading date and each of the six yield traits in the near isogenic line populations.

Population	Location	Sowing time	NP	NSP	NGP	SF	TGW	GY
R1-NIL	Lingshui	Dec. 2017	-0.004	0.823**	0.828**	0.217	0.614**	0.773**
	Hangzhou	May 2017	-0.670**	0.355*	0.147	-0.233	0.708**	-0.168
R2-NIL	Hangzhou	May 2018	-0.349**	0.806**	0.507**	-0.855**	0.672**	-0.218*

NP, number of panicles per plant; NSP, number of spikelets per panicle; NGP, number of grains per panicle; SF, spikelet fertility (%); TGW, 1000-grain weight (g); GY, grain weight per plant (g). *, $P < 0.05$; **, $P < 0.01$.
